# Most common diagnoses and antibiotics used in South American Camelid patients at a university clinic in Austria

**DOI:** 10.3389/fvets.2023.1258812

**Published:** 2023-10-06

**Authors:** Alexandra Hund, Thomas Wittek, Ursa Selan, Annemarie Käsbohrer, Clair L. Firth

**Affiliations:** ^1^University Clinic for Ruminants, University of Veterinary Medicine, Vienna, Austria; ^2^Agricultural Centre for Cattle, Grassland, Dairy, Game and Fisheries of Baden Wuerttemberg (LAZBW), Aulendorf, Germany; ^3^Unit of Veterinary Public Health & Epidemiology, University of Veterinary Medicine, Vienna, Austria

**Keywords:** South American Camelids, minor species, diagnosis, antibiotic use, EMA category, antibiotics, antimicrobial

## Abstract

Knowledge of common diseases and their treatment in minor species, such as llamas and alpacas, is growing, but frequently drugs are not licensed in these species. Our aim was to evaluate frequent diagnoses and commonly applied antibiotics in patients of a university clinic with a particular focus on EMA Category B antibiotics, which are critically important for human health. We retrospectively analyzed anonymized patient records between 2005 and 2019 regarding the causes for antibiotic treatment and choice of antibiotic substance. The most frequent indications for antibiotic treatment were diseases of the digestive tract and perioperative prophylaxis for castrations. The number of applications of EMA Category B antibiotics initially increased with the number of patients treated, then remained stable, while the use of Category D drugs increased over time. Most Category B antibiotics were used for diseases of crias and diseases of the digestive tract, primarily dental disease. The use of EMA Category B antibiotics cannot be completely avoided based on the types of cases treated. However, antibiotic stewardship guidelines should be followed wherever possible.

## 1. Introduction

The popularity of South American Camelids (SACs) is on the rise in Europe. In Austria, there were around 100 such animals in the country 30 years ago, and nowadays the total number of llamas and alpacas is estimated to be close to 10,000 (1). They are used for breeding and their fiber, but also for hiking and as trekking animals, or as therapy animals and smallholder pets (1, 2).

Their rising numbers also mean increasing demands for veterinary treatment and care. Even though camelids are foregut fermenters like ruminants, they are functionally different in many aspects (3). The body of research regarding SACs as patients is slowly growing, however, there is still limited evidence-based data on frequent diseases and drug administration (4, 5).

In the European Union and also in Austria, SACs are legally classified as farmed game (6). This means that only substances listed in (EU) regulation 37/2010 are permitted to be used. As there are no substances, including antibiotics, approved for llama and alpaca patients in Europe, every drug needs to be used off-label and rules regarding food-producing animals apply (7, 8).

As with all antibiotic treatments, prudent use to reduce the risk of antibiotic resistance (AMR) is warranted in SACs. Official guidelines exist on the general use of antibacterial substances in veterinary medicine in Austria (9), and the European Medicines Agency (EMA) recently published an updated ranking (Categories A to D) of antibiotic substances (10). They considered both the risk that their use in animals causes to public health through the possible development of antibiotic resistance and the need to use them in veterinary medicine (11).

The aim of the present study was to retrospectively evaluate which diseases led to antibiotic treatment in SACs referred to the University Clinic for Ruminants (UCR) of the University of Veterinary Medicine, Vienna, Austria. Furthermore, we assessed which antibiotic substances were most frequently used in these minor species from 2005 until 2019.

Our hypothesis was that the number of cases increased over the study period and with them the number of applications of antibiotics. However, with the rising demand for prudent use of critically important antibiotics, we anticipated that the proportion of EMA Category B applications would decrease over time.

## 2. Materials and methods

At the UCR, all patient information and medical records are entered and stored in an “animal hospital information system” (AHIS). The information pertinent to SAC was collected and exported into an Excel spreadsheet (Microsoft Excel, Microsoft Corporation, United States) for the period from January 1st, 2005 through December 31st, 2019.

SAC were admitted as patients to the UCR with a wide variety of presenting complaints. In our analysis, we included only animals with diagnoses related to antibiotic treatments. The diagnoses were classified using the Health Monitoring (*Gesundheitsmonitoring*, GMON) coding system, which is part of a nationwide health monitoring system for cattle (Supplementary Table S1) (12). Additional categories were included to account for diseases that are common in SACs and every animal was coded with a primary diagnosis and, in some cases, a secondary diagnosis. With respect to the category “diseases of crias,” we included all animals up to the age of 6 months.

Antibiotic substances used in SAC patients at the UCR were classified according to the European Medicines Agency (EMA) classification system with the four categories A to D (Table 1). In the analysis presented here, the term application refers to the administration of an individual dose to an animal. A difference may be observed between the number of patients and number of treatment courses due to some patients being treated more than once. These were then described as different treatment courses. Also, some patients received more than one antibiotic drug and were then counted multiple times in different categories.

**Table 1:** EMA categories and antibiotic substances used in this study population.

EMA Category		Antibiotic class	Substance
A – avoid	Not authorized for veterinary use in the EU	–	–
B – restrict	Critically important for human health	Cephalosporin 3rd- and 4th-generation	Cefquinome
			Ceftiofur
		Quinolones	Enrofloxacin
			Marbofloxacin
C – caution	Alternatives exist in human medicine	Aminoglycosides (except spectinomycin)	Gentamicin
			Dihydrostreptomycin (+ benzylpenicillin)
		Aminopenicillins, in combination with beta lactamase inhibitors	Amoxicillin + clavulanic acid
		Amphenicols	Florfenicol
D – prudence	First line treatments but only when medically necessary	Aminopenicillins	Amoxicillin
			Ampicillin
		Tetracyclines	Oxytetracycline
		Sulfonamides	Sulfamethoxazol/trimethoprim
		Natural, narrow-spectrum penicillins (beta lactamase-sensitive penicillins)	Benzylpenicillin

Colors have been used in accordance with the published EMA categorization (10).

Data curation and descriptive data analysis was performed using R and the packages dplyr, ggplot2, and reshape2 (13–16).

## 3. Results

### 3.1. Patient population

A total of 634 SAC patients were treated at the UCR from 2005 to 2019. Of those, 204 medical cases in 202 alpacas and 139 cases in 136 llamas were treated with antibiotic substances. Five animals were patients twice over the fifteen-year period. To account for this, we will refer to patients and cases when there is a difference. Per year, between two and 21 llamas were treated, and while the first alpaca patient requiring antibiotics was treated at the clinic in 2008, the maximum number (*n* = 33) of alpaca patients was treated in 2019. In all but one llama and seven alpacas, the sex of the animals was generally recorded in the AHIS: Over the years, more cases in male than female llamas were treated (89 entire males, 15 castrated males, 34 females), but was almost equal in alpacas (78 entire males, 25 castrated males and 94 females). Alpacas ranged in age from 1 day to 16 years, with a mean of 38.9 (± 35.7) months, whereas llamas were aged 2 days to 18 years, with a mean of 59.8 (± 51.5) months.

### 3.2. Clinical diagnoses requiring antibiotic treatment

**Figure 1:**
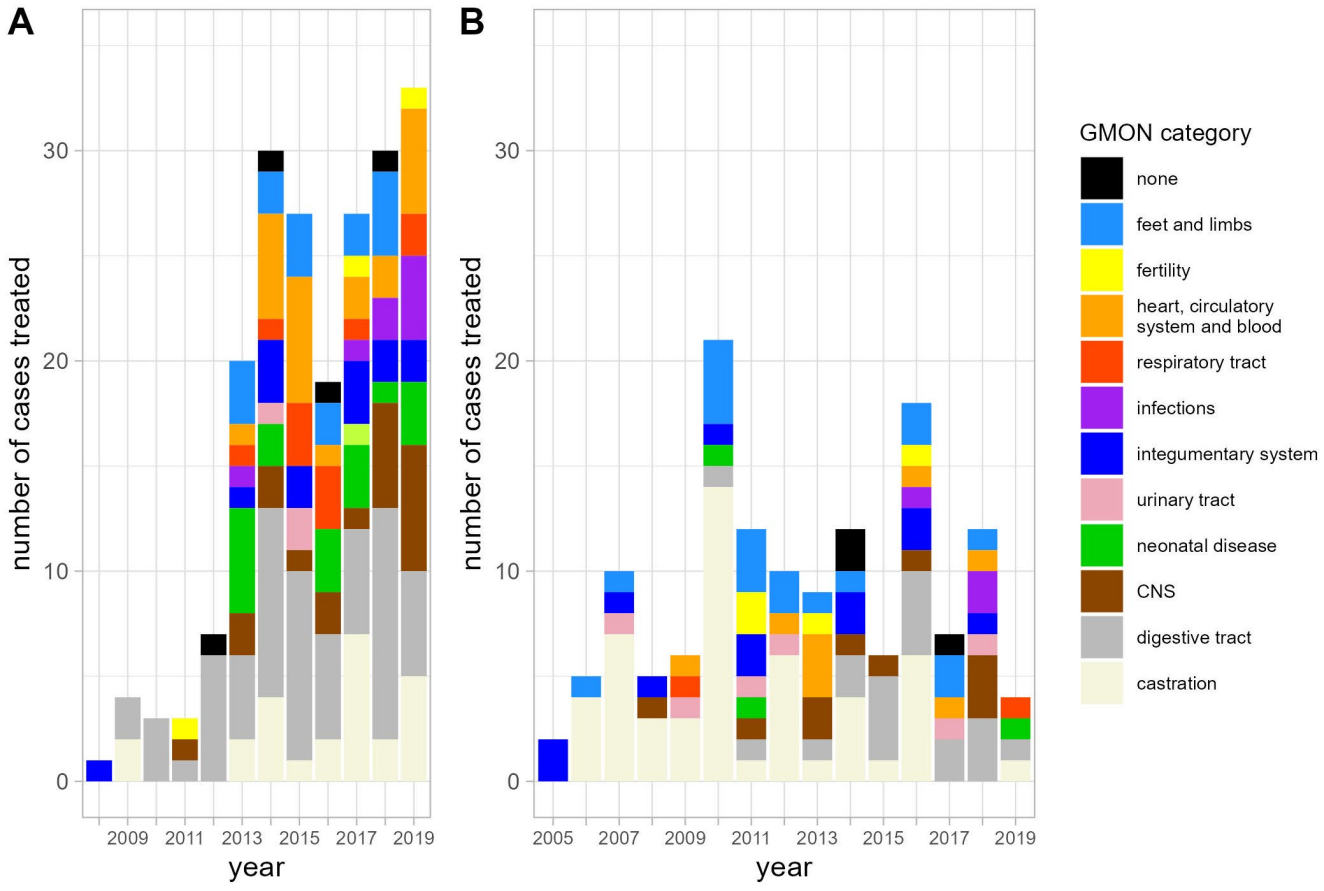
Diagnoses requiring antibiotic treatment in alpacas **(A)** and llamas **(B)** by category in the years from 2005 until 2019.

**Figure 2:**
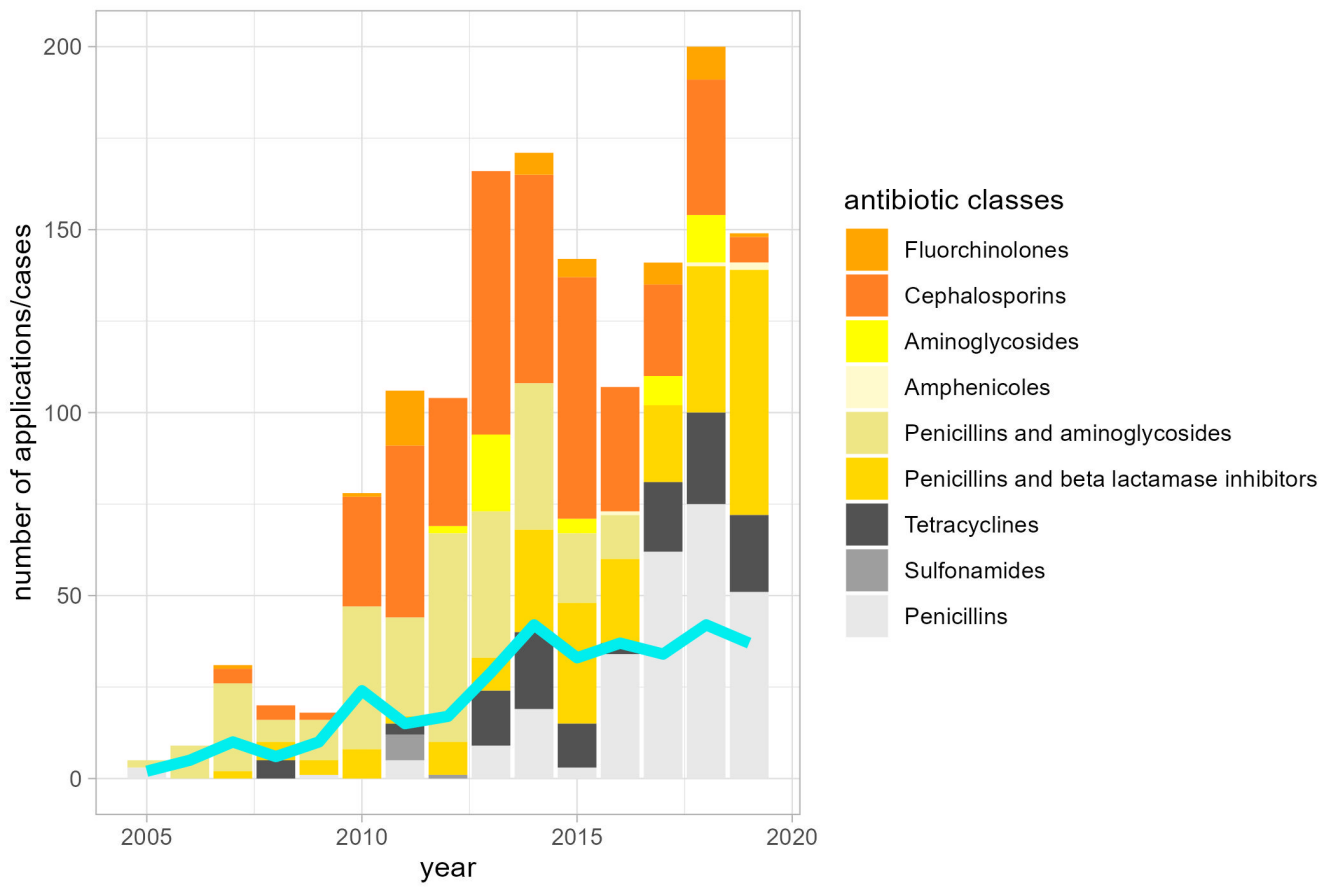
Number of applications of antibiotic classes and number of patients (turquoise line) between 2005 and 2019.

**Figure 3:**
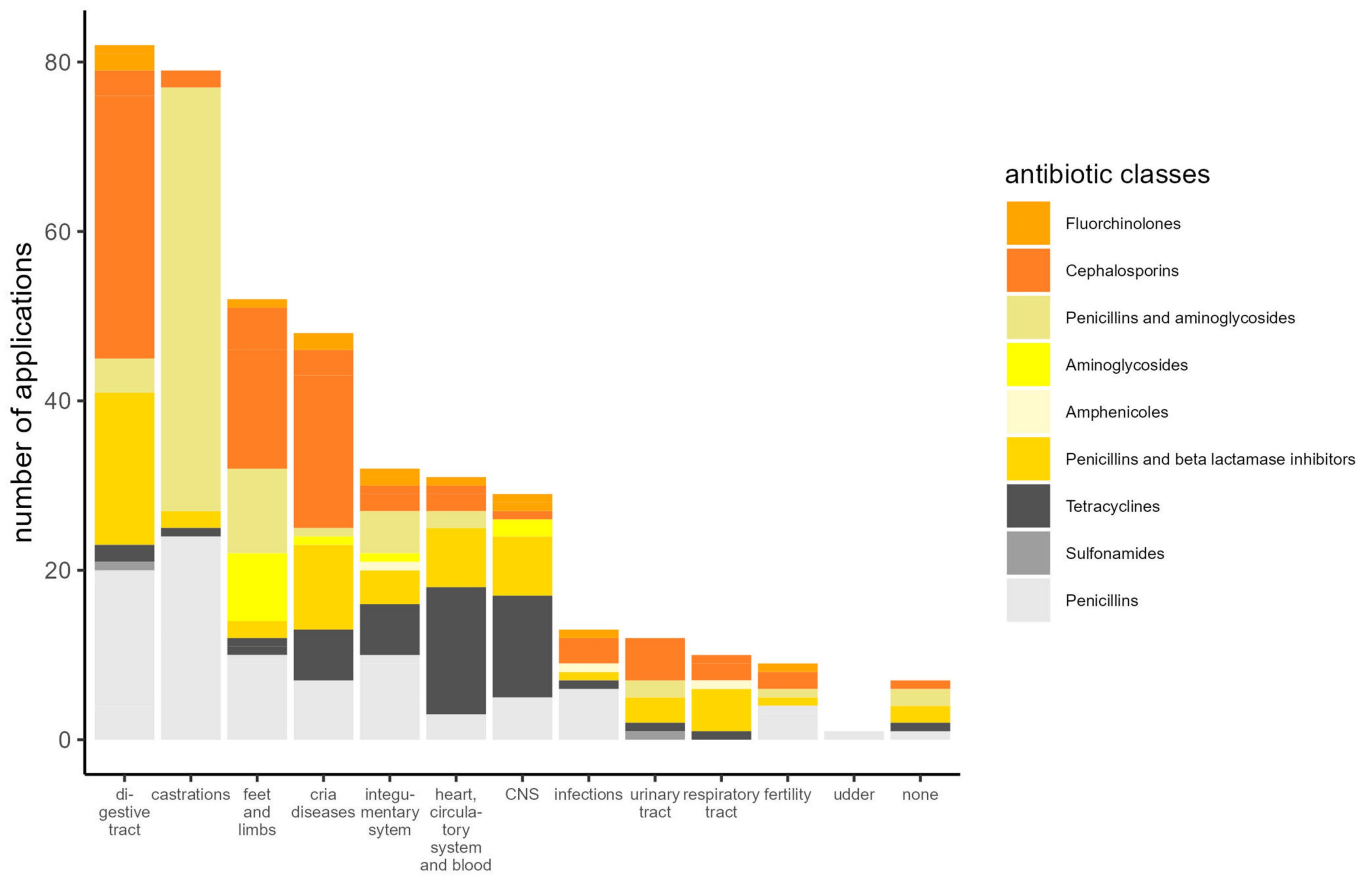
Antibiotic classes used for the treatment of diseases in SAC shown in GMON categories.

The most frequent indications for antibiotic use were diseases of the digestive tract and castrations (Figure 1). As would be expected, the number of antibiotic applications per year increased with the patient numbers (Figure 2). In the initial years of SAC patient therapy, the most commonly used substance by number of applications was a medicinal product containing both a penicillin and aminoglycoside (benzylpenicillin and dihydrostreptomycin). It was most often used in patients as perioperative prophylaxis for castration, and in orthopedic cases (Table 2; Figure 3). Third and 4th generation cephalosporins (EMA Category B) were more frequently in use starting in 2010, mainly for diseases of the digestive tract and of crias (Table 2). By the number of antibiotic treatment courses administered, the 4th generation cephalosporin, cefquinome, was most commonly used (Table 2).

**Table 2:** Number and proportion of antibiotic treatment courses by GMON diagnosis and EMA category.

		Number (%) of antibiotic treatment courses per diagnosis												
Antibiotic & number (%) of applications (*N* = 1466)	*n* treatment courses	Castrations	Cria diseases	Digestive tract	Feet and limbs	Respiratory tract	Heart, circulatory system, blood	Urinary tract	Central nervous system	Integumentary system	Infections	Fertility	Udder	None
**Cefquinome** 347 (23.7%)	83	2 (2.5%)	18 (37.5%)	31 (37.8%)	14 (26.9%)	2 (20.0%)	2 (6.5%)	5 (41.7%)	1 (3.5%)	2 (6.3%)	3 (23.1%)	2 (22.2%)		1 (14.3%)
**Ceftiofur** 73 (5.0%)	14		3 (6.3%)	3 (3.7%)	5 (9.6%)	1 (10.0%)	1 (3.2%)			1 (3.1%)				
**Enrofloxacin** 34 (2.3%)	9		2 (4.2%)	2 (2.4%)	1 (1.9%)				1 (3.5%)	1 (3.1%)	1 (7.7%)	1 (11.1%)		
**Marbofloxacin** 10 (0.7%)	3			1 (1.2%)			1 (3.2%)			1 (3.1%)				
**Ofloxacin** 1 (0.1%)	1								1 (3.5 %)					
**Amoxicillin/clav**. 251 (17.1%)	62	2 (2.5%)	10 (20.8%)	18 (22.0%)	2 (3.9%)	5 (50.0%)	7 (22.6%)	3 (25.0%)	7 (24.1%)	4 (12.5 %)	1 (7.7%)	1 (11.1%)		2 (28.6%)
**Benzylpenicillin/streptomycin** 286 (19.5%)	77	50 (63.3%)	1 (2.1%)	4 (4.9%)	10 (19.2%)		2 (6.5%)	2 (16.7%)		5 (15.6%)		1 (11.1%)		2 (28.6%)
**Florfenicol** 4 (0.3%)	3					1 (10.0%)				1 (3.1%)	1 (7.7%)			
**Gentamicin** 49 (3.3%)	12		1 (2.1%)		8 (15.4%)				2 (6.9%)	1 (3.1%)				
**Amoxicillin** 46 (3.1%)	28	12 (15.2%)	1 (2.1%)	4 (4.9%)	1 (1.9%)		1 (3.2%)		1 (3.5%)	5 (15.6%)		3 (33.3%)		
**Ampicillin** 205 (14.0%)	59	12 (15.2%)	6 (12.5%)	15 (18.3%)	8 (15.4%)		2 (6.5%)		4 (13.8%)	4 (12.5%)	6 (46.2%)		1 (100%)	1 (14.3%)
**Benzylpenicillin** 11 (0.8%)	4			1 (1.2%)	1 (1.9%)					1 (3.1%)		1 (11.1%)		
**Chlortetracycline** 2 (0.1%)	2	1 (1.3%)			1 (1.9%)									
**Oxytetracycline** 139 (9.5%)	46		6 (12.5%)	2 (2.4%)	1 (1.9%)	1 (10.0%)	15 (48.4%)	1 (8.3%)	12 (41.4%)	6 (18.8%)	1 (7.7%)			1 (14.3%)
**Sulfamethoxazol/trimethoprim** 8 (0.6%)	2			1 (1.2%)				1 (8.3%)						
Total number of treatment courses	405	79 (100%)	48 (100%)	82 (100%)	52 (100%)	10 (100%)	31 (100%)	12 (100%)	29 (100%)	32 (100%)	13 (100%)	9 (100%)	1 (100%)	7 (100%)

Please note that the total number of antibiotics applied per treatment course exceeds the number of cases because some animals were treated with more than one antibiotic drug.

EMA Category B = orange, EMA Category C = yellow, EMA Category D = grey background.

**Figure 4:**
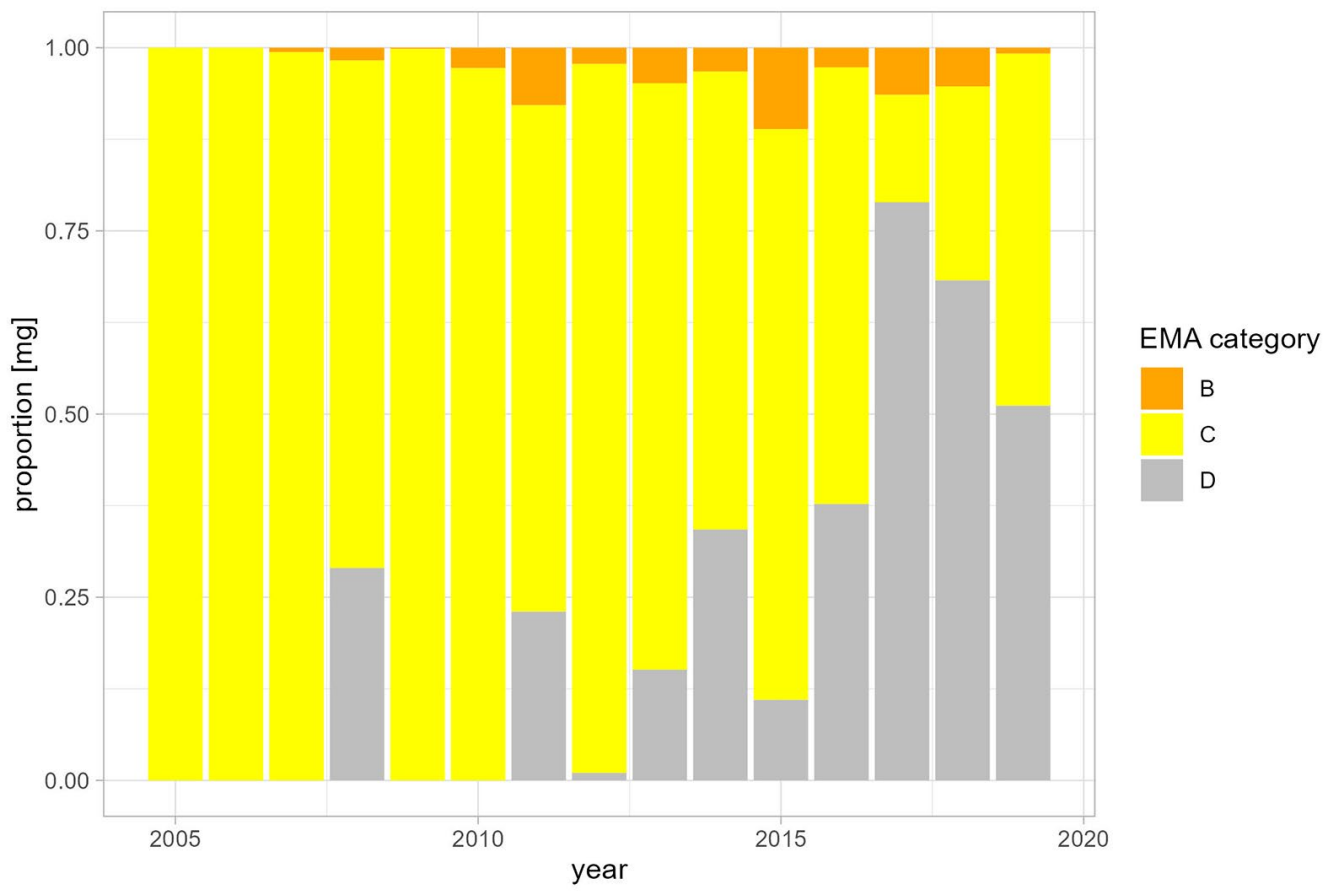
Proportion of antibiotics used in mg per year shown as EMA categories.

Over time, there was an increase in the use of EMA Category D penicillins (natural narrow-spectrum penicillins (beta lactamase sensitive) and aminopenicillins) and in the use of aminopenicillins in combination with beta-lactamase inhibitors (EMA category C). From 2017 onwards, Category D (first-line choice) antibiotics made up more than 50% of the annual antibiotic use in milligrams (Figure 4). Category B antibiotics ranged from a minimum proportion of 0% of annual antibiotic use in 2005 and 2006 rising to a maximum of 11.1% in 2015 (Figure 4). Aminoglycosides, sulfonamides and amphenicols were the least used antibiotic substances in camelid patients at the University Clinic for Ruminants over the observed study period (Table 2).

Please note that, in the following sections, only the most frequent individual diagnoses in the disease categories are described.

#### 3.2.1. Digestive tract

By number of treatment courses, the most frequent disease indication treated with antibiotics were digestive tract disorders, namely 82 treatment courses (Table 2). Around half of the patients treated antibiotically in the category diseases of the digestive tract were treated for oral disease (*n* = 38 out of 71 patients, 53.5%). Of these, one patient suffered from palatochisis, while the remainder were admitted for dental disease. The cardinal symptom was primarily local swelling, with tooth root abscesses and osteomyelitis among the most commonly observed pathologies. In 23 patients (32.3%), surgical extraction of the affected teeth was performed. In eight other dental patients, fistulating abscesses were the main causes for receiving antibiotics. Five animals were treated for diseases of the esophagus and three patients suffering from other diseases of the abdominal cavity were diagnosed with rectal prolapse (*n* = 2) or diaphragmatic hernia (*n* = 1). Antibiotics used for the treatment of all patients suffering from diseases of the digestive tract are shown in Table 2.

Twenty-five patients received antibiotic treatment based on tentative or confirmed diagnoses of *Clostridium perfringens* (*C. perfringens*) enterotoxemia. The tentative diagnosis was made in patients with rapid onset of illness that showed a combination of gastrointestinal symptoms such as signs of colic, diarrhea, intestinal gas tympani, or symptoms of central nervous system disease such as convulsions, reduced mentation or recumbency. Five patients tested positive for *C. perfringens* Type A toxin. In all of these patients, a fecal sample was taken for parasitic examination, and all samples were positive for different types and numbers of endoparasites. Overall, Category B substances, primarily the 4th generation cephalosporin, cefquinome, were most frequently applied to patients suffering from diseases affecting the digestive tract (Table 2), mainly dental treatments and those with assumed or proven *C. perfringens* infections.

#### 3.2.2. Castrations

During the observation period, the second most frequent reason for antibiotic use (with 79 treatment courses) was perioperative infection prophylaxis for surgical castration (Table 2). The age of the castrated animals ranged from 12 to 144 months, with a mean of 31.41 months. Three of these animals received the combined product of benzylpenicillin and streptomycin plus another antibiotic substance (local treatment using a 4th generation cephalosporin intramammary product or chlortetracycline spray, or a 4th generation cephalosporin systemically). Each animal received on average 2.1 (± 1.0) applications of antibiotic substances.

#### 3.2.3. Feet and limbs

Between 2005 and 2019, 33 patients received 52 antibiotic treatment courses for orthopedic problems. One animal suffered from recumbency due to diseases of the musculoskeletal system caused by traumatic gonitis and 32 patients were affected by “fractures, wounds and other injuries of the limbs.” Eleven of these (34.4%) suffered from wounds localized in the feet or limbs (secondary diagnosis) that were infected or had caused septic arthritis of different joints. In two patients, toes were amputated proximally to the medial phalanges due to purulent inflammation and osteomyelitis, and one patient was treated because of a traumatic exungulation. In cases involving joints (*n* = 8), ampicillin was often used, and gentamicin was used for infections, for example in open fractures, with suspected Gram–negative bacteria (*n* = 8) (Table 2). Ten animals received more than one antibiotic drug, in four cases this combination consisted of gentamicin and a penicillin. Overall, five patients in this group were euthanased due to grave prognoses.

#### 3.2.4. Cria diseases

During the observation period, 44 crias up to 6 months of age were treated with antibiotics, of which four animals were treated with two antibiotic substances each (i.e., 48 treatment courses in total, Table 2). These animals were treated for diseases in the GMON category “cria diseases,” but also for diseases that were classified as other GMON categories. The most frequent diagnosis was “other diseases of crias” in 16 (36.4%) animals. Twelve (75.0%) of these were neonates (less than 1 month of age) showing symptoms of weakness and/or general infections. Other frequent diagnoses in crias were diseases of the central nervous system (CNS) (*n* = 5), diseases of the sensory organs (*n* = 3) and umbilical infections (*n* = 3). Four animals were affected by disorders of the digestive tract, three animals each by respiratory disease and diseases affecting the heart, circulatory system, and blood. Out of all 44 animals in this category, 28 (63.6%) were euthanased or died.

#### 3.2.5. Integumentary system

In 32 antibiotic treatment courses in 25 animals, skin diseases were the reason for antibiotic treatment. In detail, eight patients were treated for wounds or trauma (32.0%), secondary bacterial infections due to parasitic infections or other infections of the skin were the reason for antibiotic treatment in nine patients (36.0%), and the remaining eight cases were classed as “other diseases of the skin” (32.0%). Of those, five suffered from abscesses and two from infections secondary to zinc-responsive dermatitis. Of the cases affected by parasitic infections of the skin, four animals were also diagnosed with zinc-responsive dermatitis as a secondary diagnosis. Six animals were treated with more than one antibiotic substance.

#### 3.2.6. Heart, circulatory system, and blood

Between 2005 and 2019, 27 patients received 31 antibiotic treatment courses for diseases of the circulatory system. Fifteen of these animals suffered from septicemia or anemia (55.6%) and one patient was diagnosed with endocarditis and subsequently euthanased. Of the 27 patients in this category, 19 animals (70.4%) were treated with antibiotics because of suspected infections with “Candidatus Mycoplasma haemolamae” (CMhl) as either the primary or secondary diagnosis. In the majority of animals with suspected CMhl infections, CMhl was confirmed by laboratory tests (68.4%; 13/19 animals). Ten of these 19 animals were treated using oxytetracycline and seven using an aminopenicillin. Overall, 13 of these 19 patients improved and were sent home for further treatment, of which seven had been treated with oxytetracycline and three with aminopenicillins while at the UCR.

#### 3.2.7. Central nervous system

A total of 21 patients received 29 antibiotic treatment courses for diseases of the central nervous system, of which five were treated for CNS symptoms (23.8%) and 17 for diseases of the sensory organs (81.0%). Of the latter, eyes were affected in twelve, and ears in five animals, with diagnoses such as keratoconjunctivitis, corneal ulcers and otitis. Five patients with ocular disease underwent enucleation. Five animals were treated with more than one antibiotic substance.

#### 3.2.8. Other categories

Antibiotic substances used for the treatment of SACs suffering from generalized infection including febrile conditions and tetanus, from urinary and respiratory tract infections, from fertility related and udder infections, as well as patients that received antibiotics without an obvious indication in their medical records are shown in Table 2.

### 3.3. Routes of administration

Of 1,466 total administrations of antibiotic drugs, the vast majority were applied intramuscularly (69.3%), followed by subcutaneous injection (14.0%) (Table 3).

**Table 3:** Routes of administration for all antibiotics applied.

Application route	NA	CRI	ia	im	ioc	iv	local	po	sc
Number (%) of applications	82 (5.6%)	2 (0.1%)	8 (0.6%)	1016 (69.3%)	7 (4.5%)	98 (6.7%)	47 (3.2%)	1 (0.7%)	205 (14.0%)

NA, not recorded; CRI, continuous rate infusion; ia, intra-articular; im, intramuscular; ioc, intraocular; iv, intravenous; po, per os (by mouth); sc, subcutaneous.

## 4. Discussion

The aim of this study was to describe clinical diagnoses and antibiotic therapy in SACs, as there is little information currently available in these minor species. We evaluated which diseases led to antibiotic treatment in SACs referred to the University of Veterinary Medicine, Vienna, Austria and which types of antibiotic substances were used from 2005 until 2019.

The increase in patient numbers concurs with the rising popularity of SACs in Austria and Germany and their subsequent need for veterinary care (17, 18). Yet, to our knowledge, no antibiotic substances have been officially licensed for use in SACs in the EU (19). Published materials on antibiotic substances used in SACs are only considered basic guidelines (20, 21). Therefore, it is often very difficult for veterinarians in the field to choose and administer the correct antibiotic. While our retrospective data analysis cannot (and does not aim to) provide definitive evidence in the form of a randomized controlled clinical trial, it does demonstrate the practices of veterinary specialists at the UCR over a prolonged period. It is important to note that our results regarding disease frequency may not totally reflect the situation of SAC patients in Austria because the UCR is not exclusively a primary care facility, and some patients were referred by practicing veterinarians in the field.

As the antibiotics administered over this 15 year period have been divided into their EMA Categories according to their importance to human medicine (i.e., Category B to D), we believe that this analysis is relevant to One Health. It is essential that veterinarians reduce their use of Category B antibiotics to ensure their continued efficacy in the human population. It is important to note that this retrospective data analysis covers treatments from almost 20 years ago (2005 onwards) and that the World Health Organization only published their first list of the highest priority critically important antimicrobials (HPCIAs) for human medicine that same year (22). The OIE (now WOAH) published their list of antimicrobials that were considered critically important for veterinary medicine in 2007, and the Austrian government and Austrian Chamber of Veterinarians did not publish their antibiotic treatment guidelines until 2013 (9, 23). For these historical reasons, the choice of antibiotics used between 2005 and 2019 may not always be justifiable today.

By number of applications, the most frequently used substance until 2010 was a medicinal product containing both benzylpenicillin and dihydrostreptomycin (Category C) as the combination provided a broad spectrum of antibiotic coverage. However, the situation of streptomycin with respect to antibiotic resistance (AMR) is unfavorable in bacteria commonly found in livestock (24). The product was subsequently replaced in the clinic with antibiotic substances classified as Category D, such as aminopenicillins without beta–lactamase inhibitors, tetracyclines and narrow-spectrum penicillins, as well as aminopenicillins with beta–lactamase inhibitors (Category C).

EMA Category B antibiotics, particularly 3rd and 4th generation cephalosporins were used frequently from 2010 onwards. This may be related to their frequent use for oral disease and cria diseases, which make up a considerable proportion of diseases treated at the University Clinic. The seriousness of the diseases treated, the fact that the university, as a referral clinic, often receives these camelid patients once their illnesses become chronic, and that, despite treatment, 64% of crias died or were euthanized, all explain to some extent why broad-spectrum antibiotics were often used. In our limited dataset, the influence of the number of certain diseases on the proportion of antibiotic substances used must be kept in mind.

The most common indication requiring antibiotic therapy in patients at the UCR were digestive tract disorders (including dental disease). The second largest group of patients was presented for castration and received antibiotic therapy as a part of perioperative infection prophylaxis, which has been shown to have beneficial effects in horses and is recommended in SACs (25, 26). Over the 15 year period analyzed here, antibiotic substances used in animals undergoing castration were most commonly broad spectrum drugs from EMA Categories C and D, while other authors recommended ceftiofur, procaine penicillin G, florfenicol and oxytetracycline (26–28).

The third major group were patients being treated for diseases of the circulatory system and urinary tract. Overall, our findings coincide with other studies surveying SAC health: The most commonly observed health issue identified in a survey among SAC owners in Germany were gastrointestinal parasites, together with other diseases of the digestive system, such as diarrhea and dental issues (17). The authors of a retrospective study of post-mortem examinations in Sweden also reported that the digestive system was most frequently affected, with parasitic gastroenteritis and hepatic disease being particularly common. Cardiovascular conditions, systemic disease and perinatal deaths, including abortions and fatal septicemia, were also diagnosed in Sweden. Other frequent findings were cachexia, dermatitis and diseases of the central nervous system (4).

Diseases of the digestive system, primarily dental disease, were the most commonly observed cause for antibiotic treatment in our patients, as previously reflected by other authors (17, 29–31). The etiology of dental disease in SAC has not yet been established, but common observations suggest a combination of genetic predisposition, diet and other management factors (30). As reported by Niehaus and Anderson (32), we also observed that SAC patients with dental disease concealed their symptoms relatively well and pain was generally not the primary complaint. Since medical treatment alone is not always successful, surgical treatment was the most frequently performed procedure for oral disease in our study and elsewhere (32). The choice of antibiotic should ideally be decided upon using microbiological confirmation and antibiotic susceptibility testing, but many cases of dental disease in SACs involve severe osteolytic changes with questionable validity of culture results (33). Over the 15 year period analyzed here, antibiotics with a broad spectrum of action against the bacteria commonly reported to be found in such lesions were frequently used (34).

Another type of disease of the digestive tract that warranted antibiotic treatment were animals with rapid onset of clinical signs suggesting enterotoxemia caused by *C. perfringens*. *C. perfringens* belongs to the natural gut flora but can proliferate due to changes in the gastrointestinal microbiome caused by endoparasites, inadequate diet, ulcers in the gastrointestinal tract and other diseases (35, 36). In our study, all patients with suspected enterotoxemia were also affected by and treated for endoparasites. Most animals were treated using cefquinome (EMA Category B). In retrospect, this use of a fourth generation cephalosporin may not have been justified in all cases. Antibiotic guidelines in Switzerland recommend benzylpenicillin in doses of 22.000 to 30.000 IU 1x per day s.c. for 5 to 7 days or longer (37). In any case, antimicrobial susceptibility testing should be performed before treatment, if possible, but with the fast onset of clostridial enterotoxemia, treatment sometimes requires a rapid decision. However, performing further diagnostics is especially valuable in these cases for ongoing treatment decisions, and to increase clinical knowledge on resistance levels as well as possible zoonotic potential (38).

Orthopedic diseases affecting feet and limbs in SAC patients were mostly caused by trauma in our study and elsewhere (39, 40). The most used antibacterial substance in orthopedic cases in this study was a 4th generation cephalosporin, cefquinome. Historically, cephalosporins were regarded as good perioperative antibiotic prophylactic treatment for elective orthopedic surgery (41) and a number of pharmacokinetic studies had demonstrated their bioavailability in SACs (42, 43), however, the use of higher generation cephalosporins in veterinary medicine should now be restricted due to their critical importance in human medicine (10, 11).

Over the observed period, most neonatal crias under 1 month of age were treated for suspected septicemia with clinical signs such as weakness and general infection. The most commonly observed cria problems seem to be related to environmental conditions, failure of passive transfer, together with prematurity and congenital conditions (44, 45). Davis and colleagues (46) showed that a large proportion of SAC deaths occurred in the first week of life. In our study, the majority of crias could not be treated successfully. Approximately half of the crias in our study were treated with a Category B antibiotic. A number of authors recommend treating neonatal sepsis in SACs with an antibiotic regimen with a good spectrum of activity against Gram-negative bacteria (44, 47). However, other authors have reported that approximately half of the bacterial isolates from cria patients presented for sepsis were Gram-positive bacteria (48). Unfortunately, with critically ill patients, a timely decision regarding treatment must be made and broad-spectrum coverage often seems to be the best option until bacteriological culture yields results.

Skin conditions in SACs are reported as one of the most challenging and frustrating disorders veterinarians need to deal with (49, 50). Infections and idiopathic hyperkeratosis/zinc responsive dermatosis are the most common dermatopathies in SACs (51). The majority of patients with dermatological conditions at the UCR were diagnosed with secondary bacterial infections following ectoparasite infestation. Those were mainly treated with aminopenicillins and benzylpenicillin-dihydrostreptomycin. Others recommended penicillin or trimethoprim–sulfadiazine for bacterial skin infections (52).

Several patients were admitted with symptoms of anemia, which is primarily caused by endoparasites, blood loss (e.g., gastrointestinal ulcers) or hemolysis, for example due to acute infection with CMhl (53). CMhl was diagnosed in the majority of our anemic patients, and every patient that was diagnosed with CMhl had a concurrent infection with one or more groups of internal parasites, which seems to have an impact on the severity of symptoms (54). A long acting oxytetracycline has been recommended as antibiotic drug of choice for treatment of CMhl (55). This reflects the choice made in the majority of our patients. Nevertheless, SACs may remain chronic carriers of CMhl even after receiving treatment with oxytetracycline (55).

Neurological symptoms in SAC may originate from metabolic and musculoskeletal disease aside from true neurological origin (56). One pregnant llama with neurological signs in our study showed possible pregnancy-related neurological signs, which is usually observed in sheep and goats as pregnancy toxemia, and rarely in SACs (57). Neurologic patients in our study received oxytetracycline, ampicillin or amoxicillin-clavulanic acid. An exact diagnosis of CNS disease can be difficult to obtain in SACs, leading to the use of broad-spectrum antibiotic agents to cover as many differential diagnoses as possible (58).

Overall, the number of antibiotic treatments increased with the number of patients at the clinic. However, the proportion of EMA Category B antibiotics did not decrease in recent years, most likely because of the influence of the number of patients with certain complex cases. Lastly, we found that the decision of the clinician regarding the antibiotic drug used for treatment of individual cases cannot be entirely reflected in medical records because many undocumented factors may influence this decision. The importance of prioritizing diagnostics for every case, particularly bacteriological culture and antimicrobial sensitivity testing, especially when using EMA Category B antibiotic substances, cannot be overstated. Bacteriological cultures (and subsequent sensitivity testing) were routinely carried out at the UCR in complex cases where the bacterial cause of infection was not clear, however, the severity of many conditions treated here often meant that treatment was started prior to diagnostic results being available. Including parasitology investigations into diagnostic considerations is relevant for several diseases of SAC, as parasites can be an important comorbidity factor in many diseases described in this study.

Research into the pharmacokinetics and pharmacodynamics of antibiotics commonly used in SACs is needed, particularly in the form of clinical trials which would improve our knowledge regarding the efficacy of treatments for common diseases. Continued veterinary education supported by academic research into SAC diseases is essential to ensure South American Camelid health and welfare is maintained.

Furthermore, as the antibiotics administered over this 15 year period have been divided up into their EMA Categories according to their importance to human medicine (i.e., Category B to D), we believe that this analysis is relevant to One Health. It is extremely important for human medicine that we, as veterinarians, reduce our use of Category B antibiotics to ensure their continued use and effectiveness for the human population.

## Data availability statement

The data analyzed in this study is subject to the following licenses/restrictions: consent has not been provided by the animal owners to make these data publicly available. Anonymised data are available upon reasonable request. Requests to access these datasets should be directed to alexandra.hund@lazbw.bwl.de.

## Ethics statement

Ethical approval was not required for the studies involving animals in accordance with the local legislation and institutional requirements because this analysis was carried out retrospectively in 2023, on existing patient data collected between 2005 and 2019. Between 2005 and 2019, the animals received standard treatments as chosen by a variety of veterinarians according to their clinical judgement. Informed consent was obtained from all animal owners for the treatment at the clinic at the time of hospitalization.

## Author contributions

AH: Conceptualization, Data curation, Formal analysis, Methodology, Visualization, Writing – original draft, Writing – review & editing. TW: Conceptualization, Resources, Writing – review & editing. US: Data curation, Writing – review & editing. AK: Conceptualization, Resources, Writing – review & editing. CF: Conceptualization, Formal analysis, Methodology, Project administration, Writing – original draft, Writing – review & editing.
