# Supplementary material for: Most common diagnoses and antibiotics used in South American Camelid patients at a university clinic in Austria
Source: Front Vet Sci. 2023 Oct 6;10:1258812. doi: 10.3389/fvets.2023.1258812 (PMC10588649; doi:10.3389/fvets.2023.1258812)
Supplement: Supplementary file 1 [file Table_1.DOCX]

Supplementary Material

Table S1: Modified GMON code key for South American camelids

| **GMON code** | **Category** | **Diagnosis** |
| --- | --- | --- |
| 11 | Cria disease | Umbilical infection |
| 12 | Cria disease | Umbilical hernia |
| 17 | Cria disease | Other diseases of crias |
| 171 | Castration | Castration |
| 23 | GI tract | C1 acidosis |
| 26 | GI tract | Ileus |
| 27 | GI tract | Other diseases of the abdominal cavity |
| 271 | GI tract | Peritonitis |
| 273 | GI tract | Clostridiosis |
| 28 | GI tract | Diseases of the mouth |
| 281 | GI tract | Tooth extraction |
| 29 | GI tract | Diseases of the esophagus |
| 41 | Fertility | Metritis |
| 45 | Fertility | Abortion and other problems in the gravid female |
| 46 | Fertility | Dystocia |
| 47 | Fertility | Birth injury |
| 99 | Fertility | Male reproductive organs |
| 52 | Udder | Chronic mastitis |
| 64 | Feet and limbs | Fractures, luxations/dislocations and other limb injuries |
| 68 | Feet and limbs | Recumbency due to diseases of the musculoskeletal system |
| 71 | Respiratory tract | Diseases of the upper respiratory tract |
| 72 | Respiratory tract | Pneumonia |
| 73 | Respiratory tract | Other diseases of the lungs |
| 81 | Heart, circulatory system and blood | Diseases of the heart |
| 82 | Heart, circulatory system and blood | Septicemia, anemia |
| 821 | Heart, circulatory system and blood | *‘Candidatus Mycoplasma haemolamae’* |
| 88 | Urinary tract | Urinary outflow obstructions |
| 91 | CNS | Diseases of the CNS |
| 92 | CNS | Diseases of the sensory organs |
| 93 | Integumentary system | Parasitic and other infections of the skin |
| 95 | Integumentary system | Other diseases of the skin |
| 951 | Integumentary system | Abscess |
| 952 | Integumentary system | Wound, trauma |
| 96 | Infections | Generalised infection |
| 100 | Integumentary system | Zinc-responsive dermatosis |
| 960 | Infections | Tetanus |
| 3 | Infections | Fever |
| 0 | Other | No diagnosis |
| 999 | Other | Perioperative infection prophylaxis |

Table S2: Information exported from the animal hospital information system for analysis in this study

| Name of the animal (where applicable) |
| --- |
| Species |
| Breed |
| Sex |
| Date of birth |
| Animal identification number |
| Date of admission and discharge |
| Date of drug application |
| Antibiotic class, and trade name of the drug |
| Administered dose and the route of administration |
| Initial and final diagnosis |
| Animal's condition upon discharge, e.g. cured, euthanased, etc. |
